# Dipeptidyl Peptidase-4 Inhibitors for the Potential Treatment of Brain Disorders; A Mini-Review With Special Focus on Linagliptin and Stroke

**DOI:** 10.3389/fneur.2019.00493

**Published:** 2019-05-08

**Authors:** Vladimer Darsalia, Odd Erik Johansen, Grazyna Lietzau, Thomas Nyström, Thomas Klein, Cesare Patrone

**Affiliations:** ^1^Department of Clinical Science and Education, Södersjukhuset, Internal Medicine, Karolinska Institutet, Stockholm, Sweden; ^2^Boehringer Ingelheim, Asker, Norway; ^3^Boehringer Ingelheim Pharma GmbH & Co. KG, Biberach, Germany

**Keywords:** dipeptidyl peptidase-4 inhibitors, linagliptin, stroke, stroke recovery, neuroprotection

## Abstract

Cerebral stroke is a leading cause of death and persistent disability of elderly in the world. Although stroke prevention by targeting several risk factors such as diabetes and hypertension has decreased the stroke incidence, the total number of strokes is increasing due to the population aging and new preventive therapies are needed. Moreover, post-stroke acute pharmacological strategies aimed to reduce stroke-induced brain injury have failed in clinical trials despite being effective in animal models. Finally, approximately 30% of surviving stroke patients do not recover from stroke and remain permanently dependent on supportive care in activities of daily living. Therefore, strategies to improve stroke recovery in the post-acute phase are highly needed. Linagliptin is a dipeptidyl peptidase-4 inhibitor which is clinically approved to reduce hyperglycemia in type 2 diabetes. The regulation of glycemia by dipeptidyl peptidase-4 inhibition is mainly achieved by preventing endogenous glucagon-like peptide-1 (GLP-1) degradation. Interestingly, linagliptin has also shown glycaemia-independent beneficial effects in animal models of stroke, Parkinson's disease and Alzheimer's disease. In some case the preclinical data have been supported with some clinical data. Although potentially very interesting for the development of new strategies against stroke and neurodegenerative disorders, the mode of action of linagliptin in the brain is still largely unknown and seems to occur in a GLP-1R-independent manner. The purpose of this mini-review is to summarize and discuss the recent experimental and clinical work regarding the effects of linagliptin in the central nervous system, with special emphasis on acute neuroprotection, stroke prevention and post-stroke recovery. We also highlight the main questions in this research field that need to be addressed in clinical perspective.

## Introduction

Stroke is a highly prevalent condition and a major cause of death and disabilities (1–5). Globally, 15 million people suffer a stroke every year with up to a 40% death rate (6). Of the surviving patients, up to 30% remain permanently disabled and require assistance in activities of daily living (5). In recent years, the incidence and mortality rates of stroke have significantly declined in high-income countries (4, 7). The decrease in stroke incidence is probably due to targeted intervention programs against major stroke risk factors such as type 2 diabetes (T2D), obesity, smoking, sedentary lifestyle, hypertension, and alcohol abuse (8), while the decrease in mortality rate and disability could be attributed to faster intervention by thrombolytics and/or clot removal surgery resulting in blood flow restoration which minimize stroke damage (9). However, there is a significant geographic variations of stroke burden (10), and the total number of strokes and associated disability burden have substantially increased due to the increase of global population and life expectancy (4, 7). For instance, in Europe the number of elderly is projected to increase by 35% by 2050 (11). Thus, the total number of stroke cases is unlikely to decrease, unless more advanced preventive and/or curative strategies will be developed.

Potential future approaches to reduce acute stroke damage that have been investigated over the last decades are therapeutic hypothermia and pharmacological neuroprotection (12, 13). However, neither of these strategies have seen successful translation into clinical practice. The major obstacle for this type of strategies is the rapid tissue death in ischemic core and the limited effective intervention time-window in the ischemic *penumbra* (14–16).

Pharmacological interventions of stroke aimed toward recovery to combat chronic post-stroke disabilities is also promising based on animal studies, although full translation from bench-to-bed remains to be achieved (17–20).

Recent research suggests that stroke therapeutics could be developed from diabetes research. In fact, several studies have shown that anti-diabetic drugs targeting the glucagon-like protein 1 receptor [(GLP-1-receptor agonists and dipeptidyl peptidase-4 inhibitors (DPP-4i)] can mediate anti-stroke efficacy in animal models, and has been suggested to decrease the incidence of stroke in some clinical studies [reviewed in (21, 22)]. These drugs are in clinical use for T2D and their robust safety profile suggest high potential for the possible repositioning into stroke therapies.

The aim of this review was to summarize the recent experimental and clinical data regarding the effects of DPP-4i (also named gliptins) in the central nervous system, with special emphasis on linagliptin and stroke. Specifically, we focused our discussion about the effects of DPP-4i in relation to stroke prevention, acute neuroprotection, and post-stroke recovery. We also highlighted the main gaps of knowledge that will need to be addressed in clinical perspective.

## Methods

This review is based on a literature search in Pubmed, or at the scientific conference websites of major international cardiology (e.g., ESC, ESC HF, ACC, or AHA) or diabetes (i.e., EASD or ADA) societies until Jan 31st 2019. Pubmed was searched using free-text terms and medical subject heading. A uniform search strategy was applied to Pubmed to identify the reported studies. The primary MeSH terms and keywords used were as follows: dipeptidyl peptidase 4 inhibitor, DPP IV, gliptins, linagliptin, stroke, ischemia, neuroprotection, Parkinson's, Alzheimer's, dementia, neurogenesis, and neuroplasticity. Studies were screened by title, abstract and full text.

## Linagliptin’s Pharmacology

**Figure 1:**
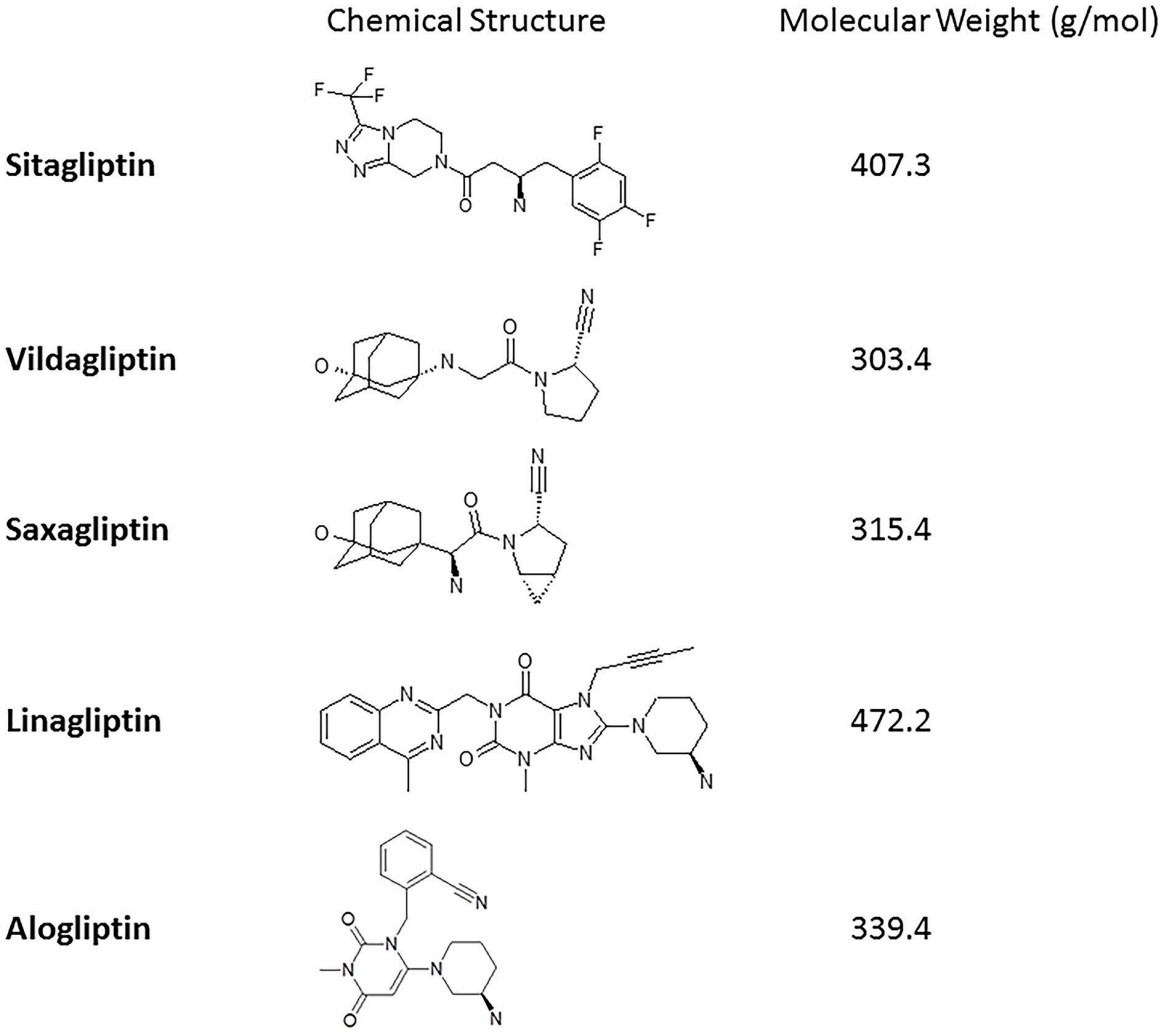
The molecular structure and weight of selected DPP-4i.

Linagliptin is a once daily oral DPP-4i launched in 2011 for the treatment of T2D. The IC_50_ of linagliptin on its primary target, DPP-4, is 1 nM, which makes it one of the most potent inhibitors within the class (23). The other DPP-4 inhibitors possess lower potency in the range of IC_50_ 7–95 nM. Linagliptin shows high selectivity for DPP-4, over other dipeptidyl peptidases and related proteases (such as DPP-8 and 9) Chemically the drug is based on an optimized and unique xanthine scaffold (See Figure 1) possessing very slow dissociation from the human DPP-4 enzyme (k_off_ < 0.00002 s^−1^). This extremely slow off-rate is the main factor for the high affinity (K_D_ = 6.6 pM) and results in a prolonged drug-target residence time over several hours (24).

Following absorption, linagliptin is distributed into tissues with high DPP-4 expression, e.g., the kidney and liver. At low concentrations (< 1 nM), 99% of linagliptin is bound to soluble and circulating DPP-4 and elimination is low. In higher concentrations (>100 nM) plasma DPP-4 is saturated and protein binding decreases to 70–80%. That is one of the reasons why in contrast to other DPP-4 inhibitors with linear pharmacokinetics, linagliptin is unique in having non-linear pharmacokinetics in the therapeutic dose (5 mg, human dose) range. The linagliptin's binding characteristics were shown to be absent in DPP-4 deficient animals (25). Moreover, renal excretion of linagliptin at its therapeutic dose is < 7%, which is unique in the DPP-4 inhibitor class, that are primarily eliminated via the kidney (26). Linagliptin further shows low interaction with Cytochrom P450 and high stability in human cytosolic and microsomal compartment, however linagliptin is a P-gp (P-glycoprotein) substrate (27) limiting penetration across the blood brain barrier (bbb) under normal conditions (28).

## Linagliptin and the Treatment of T2D

The concept behind DPP-4i for therapeutic use in T2D is based on the prolongation of the half-life of the incretins GLP-1 and glucose-dependent insulinotropic polypeptide (GIP), both secreted via specific endocrine gut cells following meal digestion. Incretins cause subsequently glucose-dependent insulin secretion for mainly postprandial glucose regulation. Further, DPP-4 inhibition suppresses glucagon secretion from α-cells. Numerous other substrates for DPP-4 have been described (29) such as SDF1α, GLP-2, NPY, substance P, which are activated or deactivated by the DPP-4 protease and play additional roles in inflammation, food intake, pain and vascular regulation, however less contribute to glucose control. Most clinically used DPP-4 inhibitors, like linagliptin, are once daily drugs because they show >80% inhibition of DPP-4 activity over 24 h. This is associated with the high increase of plasma GLP-1 and consistent reductions in elevated plasma glucose and HbA1c in various patient populations and across various background therapies (30). The recently completed cardiovascular outcome trial CARMELINA (CArdiovascular and Renal Microvascular outcomE study with LINAgliptin) confirmed the tolerability of linagliptin, and its cardiovascular (CV) safety, without any signal of heart failure (31). Due to an excretion primarily via the bile, linagliptin does not need dose adjustment, including in patients with T2D and impaired kidney function. CARMELINA proved safety in these renally impaired patients, and additionally showed a significant reduction in risk for progression albuminuria (32), which has not been assessed in a similar robust manner with the other members of the class of DPP-4 inhibitors.

## Linagliptin Mediates Beneficial Effects in Experimental Models of Brain Disorders

Beside their glycemic properties, it has been recently reported that DPP-4 inhibitors can also affect the brain. For instance, studies have shown that DPP-4i exert neuroprotective actions in animal models of Parkinson's disease (PD) (33–35) and ongoing studies investigating the safety of intranasal delivery of the DPP-4i omarigliptin for the treatment of PD are ongoing (36). Moreover, D'Amico et al. (37) have shown that sitagliptin delayed AD-like pathology in a mouse model, and several studies confirmed these findings by using different DPP-4i inhibitors, including linagliptin [(38–41) and reviewed in (42)]. Other effects improving cognitive function, neuroplasticity and neurogenesis have been recently reported by employing vildagliptin (43), sitagliptin (44–48), and linagliptin (49). Furthermore, Hasegawa et al. (50) showed that linagliptin decreased hippocampal neuronal cell death and improved cognitive function in a model of aging. However, one study has also shown negative effects of sitagliptin in the brain, i.e., increased tau phosphorylation and insulin resistance (51). Finally, a recent study showed neuroprotection by sitagliptin in a model of brain trauma (52). In summary, although the passage of DPP-4i through the bbb in the damaged brain is undetermined and the mechanisms are unknown, the evidence of favorable effects of DPP-4i on brain complications is substantial.

## Linagliptin Provides Acute Neuroprotection After Stroke in Experimental Models

It is difficult to study the potential efficacy of candidate drugs to reduce stroke risk using animal models. However, to determine the efficacy for acute neuroprotection, several animal models exist. A few studies have tested the potential efficacy of DPP-4i for acute neuroprotection and/or recovery after stroke. Moreover, acute ischemic stroke severity has been recently associated to changes in DPP-4 activity (53), suggesting that the regulation of this enzyme might have a therapeutic value.

Rohnert et al. (54) first showed that DPP-4 inhibition is neuroprotective in stroke via intracerebral administration of sitagliptin in the rat. We recently showed that 4 weeks per-oral pretreatment followed by 3 weeks post-stroke treatment with linagliptin reduced brain damage after stroke, in both normal and T2D/obese mice (55). Similar effects in non-diabetic rats were recently shown by Yang et al. (56) using alogliptin and by El-Sahar et al. (57) using vildagliptin. In the Yang et al. study, neuroprotection correlated to increased levels of brain BDNF. Moreover, DPP-4 inhibition by genistein resulted in similar findings (58). By using an experimental design consisting of chronic administration of linagliptin before and after stroke, Darsalia et al. (59) also showed that linagliptin-mediated neuroprotection against stroke occurred in correlation with increased neural stem cells proliferation (60) and, importantly, was not mediated by the GLP-1R.

A recent work by Ma et al. (61) has shown that the linagliptin treatment starting after stroke can decrease the stroke-induced brain damage in a rat model of transient cerebral ischemia induced by bilateral common carotid artery occlusion. Similar data using transient middle cerebral occlusion were reported by Chiazza et al. (62) who also showed that linagliptin improved functional recovery 3 days after stroke through the activation of the SDF-1α/CXCR4 pathway. Although one cannot rule out that the results of these two studies are due to the presence of linagliptin close to stroke time (suggesting acute neuroprotective effects), the data also suggest a pharmacological effect that goes beyond acute neuroprotection because the study design allowed extending the observation period from days to weeks after experimental stroke, thus evaluating the effects of linagliptin treatment in the post-stroke recovery phase. The likelihood of positive effects during the post-stroke recovery phase is also supported by the work of Darsalia et al. (59) showing that a single, acute *bolus* administration of linagliptin at stroke time was ineffective in reducing the brain damage. Furthermore, the potential role of DPP-4 inhibition in endogenous brain tissue remodeling and repair processes after stroke has been recently suggested by Wesley et al. (63).

Whether DPP-4i leads to neuroprotection and promotes recovery after stroke by directly acting on neurons remains to be determined, although a recent *in vitro* study suggested direct neuroprotection in neural cells (64). However, additional cellular mechanisms may be involved. Indeed, Mi et al. (65) showed that linagliptin increases the *in vitro* proliferation of rat brain microvascular endothelial cells via the SIRT1/HIF-1α/VEGF pathway. Furthermore, recent works have shown that linagliptin improves cerebrovascular dysfunction and remodeling in a rat model of T2D, independent of glycemic control (66, 67).

Admission hyperglycemia is *per se* a negative prognostic marker for patients suffering acute ischemic stroke. Interestingly, hyperglycemia in patients without previously known diabetes is associated with a greater risk for poor outcome compared to patients with identified diabetes prior the stroke (68–70). Remarkably most of the effects reviewed here occurred independently from the regulation of glycemia in both normal and diabetic models, suggesting that the potential efficacy of DPP-4i to increase acute neuroprotection and/or recovery may be clinically relevant not only for the diabetic population under a DPP-4i-mediated therapy.

## DPP-4 Inhibitors and Stroke Prevention With a Focus on CARMELINA, a Cardiorenal Outcome Trial With Linagliptin

The incidence of CV complications in T2D, including stroke, has declined substantially but it is still high (71). An increasing CV risk factor control attainment has probably contributed to this, and recently, it was demonstrated that maintaining control of glycated hemoglobin level, low-density lipoprotein cholesterol level, albuminuria, and blood pressure, as well as abstaining from smoking, was associated with no excess risk of CV death or stroke in T2D as compared to the general population (72). Interestingly, glycated hemoglobin was the strongest risk factor for stroke in this registry analysis.

Because, historically, there have been CV safety concerns regarding anti-diabetic drugs in the treatment of T2D, regulators in US and Europe focus specifically on this issue [(73) and http://www.ema.europa.eu/docs/en_GB/document_library/Scientific_guideline/2012/06/WC500129256.pdf], and as a response, fifteen CV outcome trials assessing 3 novel classes of antihyperglycemic therapies (i.e., DPP-4 inhibitors, GLP-1 receptor agonists, and SGLT-2 inhibitors) had been completed by end of 2018, of which none reported an increase in risk for major adverse CV events (MACE), whereas 6 agents have demonstrated CV benefits (74). Within the class of DPP-4i, four large CV outcome trials have been published till date for saxagliptin (75), sitagliptin (76), alogliptin (77), and linagliptin (32).

These trials have used the composite Major Adverse CV events (MACE); i.e., CV death, non-fatal myocardial infarction and stroke, with or without hospitalized unstable angina, as primary outcome. All trials have demonstrated CV safety, without incremental benefit for MACE, including no statistical significant difference in risk for non-fatal stroke. In the saxagliptin trial there was however a significantly higher numbers of patients hospitalized for heart failure (75), a signal also reported in the alogliptin trial (77). Due to this, the class has received a heart failure warning by the US Food and Drug Administration, especially if used in patients with high CV risk underlying heart and kidney disease (73). The linagliptin trial (CARMELINA^®^) (32) was designed to evaluate the CV safety and kidney outcomes of linagliptin in patients with T2D at high CV risk (75% of patients had prevalent kidney disease). Despite a frailer population, in comparison with the other DPP4i trials, linagliptin resulted in a non-inferior risk of MACE [compared to placebo added to standard care; hazard ratio 1.02 (95% CI: 0.89, 1.17)], including across a number of subgroups such as by sex and age, and did not affect the risk of heart failure [hazard ratio 0.90 (95% CI: 0.74, 1.08)] (31). Furthermore, the progression of albuminuria occurred less frequently in the linagliptin group [hazard ratio 0.86 (95% CI: 0.78, 0.95)] (32), but despite MACE safety, no significant protection for stroke [fatal/non-fatal stroke hazard ratio 0.91 (95% CI: 0.67, 1.23)] was observed. Nonetheless, their broad tolerability and the safety profile are today well-documented and a good choice for the pharmacology treatment of T2D. Importantly, it remains to be determined if these class of drugs can improve stroke outcome in the recovery phase (78) as it has been shown in several experimental studies. Interestingly, outcome trials with linagliptin (32, 79) will be exploring this question by determining the post-stroke functional outcome in a subgroup of patients hospitalized with stroke with T2D by using the modified rankin scale 3–6 months after stroke.

## Linagliptin and Clinical Effects on Cognitive Outcomes

Cognitive impairment, including mild cognitive impairment (MCI) and dementia, is increasingly recognized as an important T2D complication (80). High HbA1C concentration and glucose variability are negatively associated with subtle cognitive changes but the association is weak and more randomized controlled trials are needed (81). The underlying processes of cognitive dysfunction in T2D are largely unknown and till date, no pharmacological intervention has proven efficacious (82). Given that incretin therapies have emerged as a potential therapeutic lead for vascular brain injury, studying effects of these on cognitive outcomes are of interest. This is further supported by findings, in an observational study in elderly patients with T2D, that increased plasma DPP-4 activity is associated with elevated risk of MCI (83) and some small, and hypothesis generating, and underpowered, clinical observational studies, reporting some benefits of DPP-4i on clinical cognitive outcomes (84–86).

Both in CARMELINA, and in CAROLINA (a recently completed head-to-head study of linagliptin vs. the sulfonylurea glimepiride) cognitive studies with linagliptin have been completed, but none yet published. The CARMELINA-cognition and the CAROLINA-cognition sub-studies were integral parts of CARMELINA (32) and CAROLINA trials (87), respectively. Both cognition sub-studies aimed to test whether linagliptin prevents accelerated cognitive decline by applying the mini-mental state examination, as a measure of global cognitive function. In addition, more domain-sensitive composite measure of attention & executive functioning, using two additional tests: the Trail Making Test and the verbal fluency test, have been applied. The results of these sub-studies will be informative for refining the research area within this emerging and highly developing field.

## Future Directions

Preclinical studies showing favorable effects of DPP-4i in several CNS disorders and stroke, encourage further research aiming to clinically reposition these diabetic drugs as active CNS-centric drugs. Clinical studies mainly investigating the safety of DPP4i have shown that these drugs are safe albeit no effect to decrease stroke incidence has been shown.

The clinical efficacy of acute neuroprotection after stroke is largely dependent on timely intervention within a very short therapeutic window (few hours from stroke onset), which is difficult to achieve. However, if the neuroprotective substance is systemically present at stroke time, the chances of minimizing stroke-induced tissue loss are significantly greater. The success of this strategy could be achieved in T2D patients (at high stroke risk) who routinely take DPP-4i for the daily management of T2D. These patients could benefit from this treatment when suffering from stroke.

Another potential strategy to exploit the advantages of a DPP4i-based stroke therapy could be the promotion of stroke recovery and rehabilitation in the post-acute/chronic phase that could be theoretically applicable to all stroke patients. However, the potential of this approach has not yet been thoroughly investigated in animal studies and research in this field is highly needed, also in the clinical setting (78), including exploring whether there are differences in effects according to patient-characteristics; a field of emerging importance in the personalized medicine area. Indeed recent animal studies have shown pro-neurogenic (46), anti-inflammatory (54) and neuroplasticity (49) effects mediated by DPP-4i that could result beneficial in the post-stroke recovery phase and long-term clinical outcome.

Finally, studies are needed to understand the molecular mechanisms of DPP-4i in the brain, since the passage of DPP-4i through the bbb seems not to occur under normal conditions and after stroke is undetermined. Therefore, to study the mechanism of action of DPP4i in the brain substances with bbb permeability need to be synthesized in the future. Moreover, the systemic peripheral doses of GIP and GLP-1 after DPP-4i administration are low in comparison to GLP-1 agonists, e.g., in the pg range, and neuroprotection by linagliptin treatment has been shown to occur independently from GLP-1R (59). This suggest that alternative mechanisms are likely involved. Since the DPP-4 is an enzyme with over 40 known, biologically active substrates, many of which with proven CNS effects, the deeper understanding of how exactly DPP-4i regulate these substrates could lead to the identifications of new therapeutic targets in the CNS.

## Conclusions

**Figure 2:**
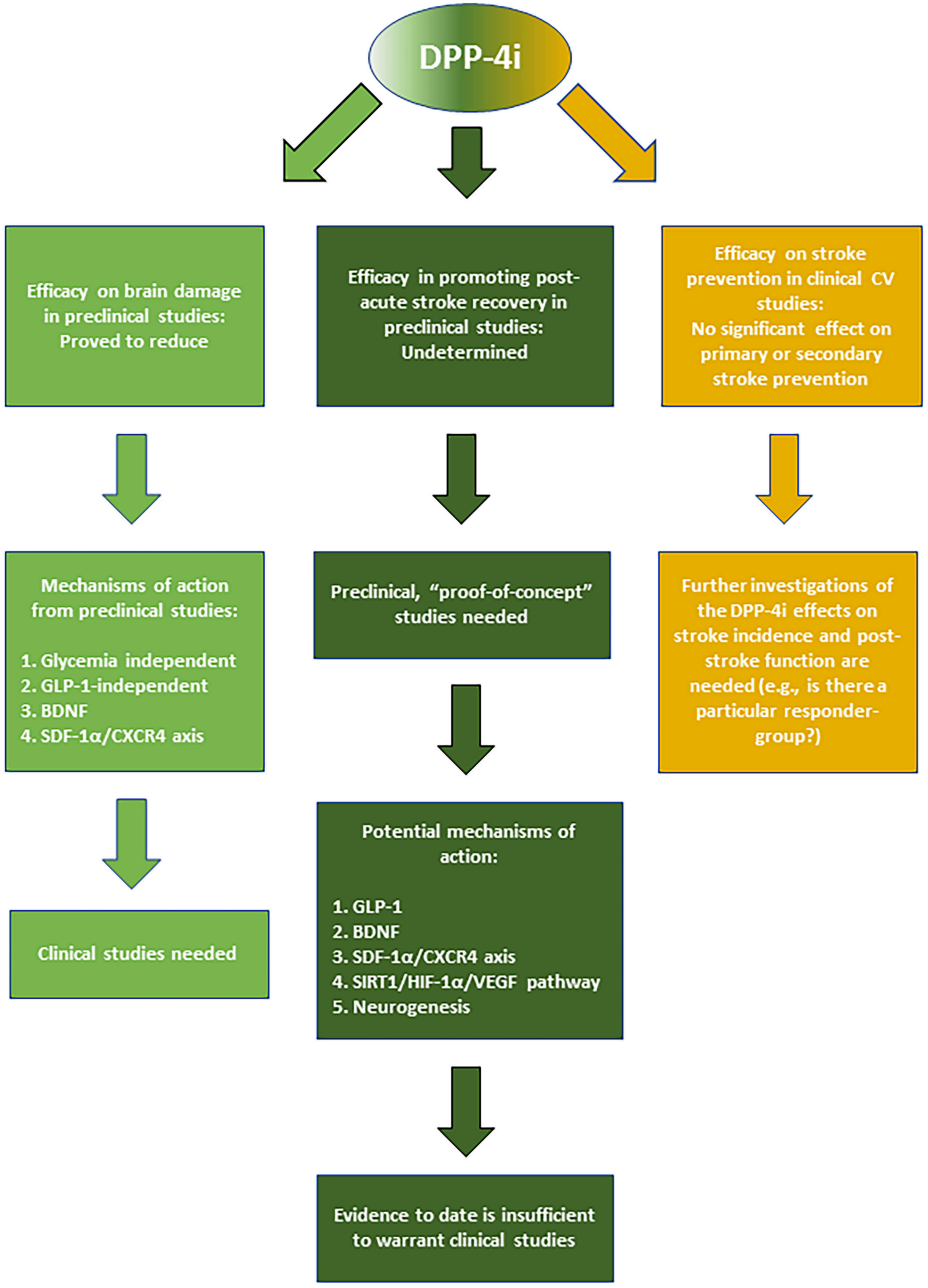
Summary of the reported effects and mechanisms mediated by DPP-4i in stroke prevention, acute neuroprotection, and post-stroke recovery.

The demonstrated beneficial CNS effects in preclinical studies and the proven clinical safety of DPP-4i make them good candidates for their potential repositioning against stroke. While the data so far suggest that DPP-4i cannot reduce stroke risk, studies are needed to determine if T2D people who use them for daily T2D management could gain advantages in terms of reduced brain damage in the event of stroke. Both T2D and non-diabetic stroke patients could also benefit from the use of DPP-4i as post-stroke curative agents promoting recovery and rehabilitation in post-acute phase. However, more pre-clinical and clinical research is highly needed in this research field (See Figure 2 for the summary of DPP-4i-mediated effects in stroke and potential mechanisms of action).

## Author Contributions

VD conceived and wrote the review. OJ wrote the Linagliptin and Clinical Effects on Cognitive Outcomes section and edited the manuscript. GL wrote the Introduction section. TN wrote and reviewed the DPP-4 Inhibitors and Stroke Prevention With a Focus on CARMELINA, a Cardiorenal Outcome Trial With Linagliptin section. TK conceived the review and wrote The Pharmacology of Linagliptin and Linagliptin and the Treatment of T2D sections. CP conceived, wrote, and coordinated the review.

### Conflict of Interest Statement

Work in our laboratory is partly financed by Boehringer Ingelheim Pharma GmbH & Co. TK is employed at Boehringer Ingelheim Pharma GmbH & Co and Odd Erik Johansen by Boehringer Ingelheim Norway. TN has received unrestricted grants from AstraZenca and consultancy fees from Boehringer Ingelheim, Eli Lilly, Novo Nordisk, Merck, and Sanofi-Aventis. The remaining authors declare that the research was conducted in the absence of any commercial or financial relationships that could be construed as a potential conflict of interest.
